# High Albumin Level Is Associated With Regression of Glucose Metabolism Disorders Upon Resolution of Acute Liver Inflammation in Hepatitis B-Related Cirrhosis

**DOI:** 10.3389/fcimb.2022.721138

**Published:** 2022-02-22

**Authors:** Caiyun Tian, Yanping Zhu, Yujuan Liu, Han Hu, Qijiao Cheng, Fangwan Yang, Lingqi Pei, Yihong Zhou, Ying Li, Shide Lin

**Affiliations:** ^1^ Department of Infectious Diseases, Affiliated Hospital of Zunyi Medical University, Zunyi, China; ^2^ College of Laboratory Medicine, Affiliated Hospital of Zunyi Medical University, Zunyi, China

**Keywords:** chronic hepatitis B, liver cirrhosis, hepatitis flare, diabetes, stress-induced hyperglycemia, pancreatic β-cell function, insulin resistance

## Abstract

**Background and Aim:**

To investigate the short-term dynamic changes and the factors associated with regression of glucose metabolism disorders in patients with hepatitis flare of chronic hepatitis B virus (HBV) infection.

**Methods:**

In this study, 118 patients with severe hepatitis flare of chronic HBV infection were prospectively studied. Oral glucose tolerance test was performed on admission and during follow-up to evaluate dynamic changes in glucose metabolism disorders. The factors associated with regression of glucose metabolism disorders were identified using univariate and multivariate logistic regression analyses.

**Results:**

The prevalence of diabetes was significantly higher in 70 (47.1%) patients with liver cirrhosis than that in 48 (16.8%) patients without liver cirrhosis. The prevalence of impaired glucose tolerance in patients with liver cirrhosis (35.7%) was significantly lower than that in patients without liver cirrhosis (47.8%). After a follow-up of 20.0 ± 18.7 days, 28 of 31 (90.3%) patients without liver cirrhosis experienced regression of glucose metabolism disorders. Additionally, 30 (54.5%) patients with liver cirrhosis experienced regression of glucose metabolism disorders after 42.0 ± 36.2 days. In patients with liver cirrhosis, those with regression of glucose metabolism disorders had significantly higher levels of homeostasis model assessment-β-cell function, albumin (ALB), and a significantly lower level of fibrosis-4 score. ALB was identified as an independent factor associated with the regression of glucose metabolism disorders in patients with liver cirrhosis.

**Conclusion:**

Severe acute liver inflammation aggravates glucose metabolism disorders in patients with hepatitis B-related liver cirrhosis and high ALB level is associated with regression of glucose metabolism disorders upon resolution of acute liver inflammation.

## Introduction

Glucose metabolism is easily altered in patients with various acute and chronic diseases, resulting in hypoglycemia, impaired glucose tolerance (IGT), or diabetes (Hamed et al., 2019). As the liver is the major organ responsible for glucose metabolism, disorders of glucose metabolism are prevalent in patients with liver diseases. Diabetes secondary to severe impairment of liver function is recognized as hepatogenous diabetes (HD); however, it is not classified as an independent disease by the World Health Organization (Orsi et al., 2016). Hyperglycemia induced by severe acute diseases is defined as stress-induced hyperglycemia (Dungan et al., 2009).

Previous studies have comprehensively investigated HD in patients with liver cirrhosis (Imazeki et al., 2008). The etiology and severity of liver cirrhosis are associated with the development of HD. Additionally, hyperglycemia has been found in patients with acute hepatitis and acute liver failure, suggesting that acute liver injury may contribute to the development of glucose metabolism disorders in patients with liver diseases.

Patients with chronic hepatitis B virus (HBV) infection are common in China. Patients with chronic HBV infection frequently experience different degrees of hepatitis flare, resulting in liver fibrosis and even liver cirrhosis. Severe flares of chronic HBV infection are at an elevated risk of further deterioration into acute-on-chronic liver failure (ACLF). Therefore, glucose metabolism disorders may differ among patients with hepatitis flare of chronic HBV infection, liver cirrhosis, or acute hepatitis. In patients with hepatitis flare of chronic HBV infection, both acute and chronic liver injuries may induce HD, and acute and chronic liver inflammation may cause stress-induced hyperglycemia. Upon resolution of acute liver inflammation and repair of acute liver injury, glucose metabolism disorders should partly regress. However, the dynamic changes and the factors associated with the regression of glucose metabolism disorders in patients with hepatitis flare of chronic HBV infection have yet to be investigated.

Both hypoglycemia and hyperglycemia have been found to be closely related with high mortality in patients with various severe diseases (Lee Y. S., et al., 2019; Kushimoto et al., 2020). According to previous studies, diabetes is associated with poor long-term prognosis and high risk of complications, such as bacterial infection, bleeding esophageal varices, hepatic encephalopathy, and hepatocellular carcinoma, in patients with liver cirrhosis (Sigal et al., 2006; Konishi et al., 2009; Erice et al., 2012; Jeon et al., 2013; Elkrief et al., 2014). These findings suggest the necessity of long-term management of glucose metabolism disorders in patients with liver cirrhosis. Therefore, the dynamic changes in glucose metabolism disorders in patients with liver cirrhosis and acute liver inflammation need to be elucidated. In this study, we explored the short-term dynamic changes of glucose metabolism disorders in patients with chronic HBV infection and severe hepatitis flare.

## Patients and Methods

### Study Participants

To investigate glucose metabolism disorders in this study, 248 patients with severe hepatitis flare of chronic HBV infection who had been hospitalized at the Department of Infectious Diseases, Affiliated Hospital of Zunyi Medical University (Zunyi, China) from October 2017 to December 2020 were prospectively recruited to undergo oral glucose tolerance test (OGTT) on admission. Thirty-four patients were excluded due to comorbidity with other liver diseases: 12 patients with alcoholic liver disease; seven patients with drug-induced hepatitis; nine patients with hepatocellular carcinoma; three patients with previous diagnosis of type 2 diabetes; three patients with previous or present consumption of steroids. Ninety-six patients with ACLF on admission or progression to ACLF after admission were also excluded. Finally, 118 patients were recruited in the study.

### Diagnostic Criteria of Severe Hepatitis Flare of Chronic HBV Infection, ACLF, Liver Cirrhosis, and Glucose Metabolism Disorders

The diagnostic criteria for severe hepatitis flare of chronic HBV infection were based on those of acute exacerbation, which were proposed by Lok and Lai (1990) and Gao et al. (2015), with minor modifications. The criteria for acute exacerbation of chronic HBV infection were as follows: (1) presence of hepatitis B surface antigen and HBV DNA for >6 months before hospitalization; (2) alanine aminotransferase (ALT) >5× the upper limit of the normal value (ULN, 200 IU/L); and (3) total bilirubin (TBil) ≥3× ULN (51 μmol/L) or prothrombin activity (PTA) of 40–60%.

ACLF was diagnosed as a recent development of jaundice (TBil ≥5×ULN) and coagulopathy (PTA <40% or international normalized ratio [INR] ≥1.5), complicated within 4 weeks by ascites and/or encephalopathy in patients with previously diagnosed or undiagnosed chronic liver disease (Sarin et al., 2019).

Liver cirrhosis was diagnosed based on previous liver biopsy findings, ultrasonography, computed tomography, liver stiffness measurement, or magnetic resonance imaging findings. Patients with irregular and nodular liver, small and shrunken liver, splenomegaly and hypersplenism, or evidence of portosystemic collaterals together with impaired liver synthetic function were diagnosed with liver cirrhosis (Tsochatzis et al., 2014).

Fasting plasma glucose (FPG), fasting insulin (FINS), and fasting C-peptide (FCP) were measured within 24 hours after admission. OGTT was performed as recommended by the World Health Organization with oral hydroglucose (75g load). Glycemia was measured at two time points: 0 and 2 hours. Glucose abnormalities were diagnosed based on American Diabetes Association criteria (Handelsman et al., 2015). A diagnosis of diabetes was established in the presence of FPG ≥7 mmol/l or 2-hour post-OGTT plasma glucose (2h-PG) ≥11.1 mmol/l. IGT included patients with impaired fasting glucose (FPG of ≥5.6 mmol/l but <7 mmol/l) and/or impaired glucose tolerance (2h-PG of ≥7.8 mmol/l and <11.1 mmol/l). Since glycated hemoglobin (HbA1c) levels may be inappropriately normal in patients with liver cirrhosis, as a result of the reduced lifespan of erythrocytes due to hypersplenism (Orsi et al., 2016), HbA1c was not included in this study.

The homeostasis model of assessment-insulin resistance (HOMA-IR) was calculated according to the following formula: FPG (mmol/L) × FINS (uIU/mL)/22.5. HOMA–insulin sensitivity (HOMA-IS) was calculated as 1/HOMA-IR. HOMA-β-cell function (HOMA-β) indices were estimated as 20×FINS (uIU/mL)/[FPG (mmol/L)-3.5] (%) (Levy et al., 1998). The severity of liver disease was assessed using the model for end-stage liver disease (MELD) score. The MELD score was calculated based on the following formula: MELD score = 3.78 × ln [TBil (mg/dL)] + 11.2 × ln [INR] + 9.57 × ln [creatinine (Cr, mg/dL)] + 6.43 × (constant for liver disease etiology = 0, if cholestatic or alcoholic, otherwise = 1).

In addition, the levels of serum markers of liver fibrosis were calculated according to the following formulae: aminotransferase (AST)-to-platelet (PLT) ratio index (APRI) = (AST/ULN) × (100/PLT); FIB-4 = (age × AST)/(PLT × square root of ALT).

### Therapeutic Methods and Follow-Up

After admission, all patients received intensive supportive treatments, such as liver protection, albumin supplementation, water and electrolyte balance maintenance, and plasma transfusion. All patients received antiviral therapy (e.g., tenofovir, entecavir, lamivudine) within three days after admission.

For patients with diabetes, oral hypoglycemic agents or insulin was initiated, if diet therapy was not sufficient to achieve good glycemic control. For patients with IGT, no specific treatment was administered. In 86 patients with diabetes or IGT without oral hypoglycemic agents or insulin treatment, OGTT was repeated to evaluate the regression of glucose metabolism disorders. Based on the OGTT results at baseline and the times of follow-up, patients were retrospectively assigned to one of the two outcome groups — (1) regression: from diabetes or IGT to normal glucose tolerance (NGT) or from diabetes to IGT; (2) non-regression: no change in glucose metabolism disorders or progression from IGT or NGT to diabetes or from NGT to IGT.

The protocol conformed to the provisions of the Declaration of Helsinki and was approved by the Human Ethics Committee of the Affiliated Hospital of Zunyi Medical University. All patients consented to the use of their data for clinical research after they were informed in writing.

### Statistical Analysis

SPSS version 19.0 software (IBM Corp., Armonk, NY, USA) was used for all statistical analyses. For normally distributed and non-normally distributed continuous data, differences between groups were assessed using unpaired t-tests and Mann Whitney U-tests, respectively. The chi-squared test was used for categorical data. Logistic regression analysis was used to determine the factors associated with the regression of glucose metabolism disorders. Univariate logistic regression analysis was first used to screen candidate factors. Candidate variables (P<0.05) entered into a multivariate logistic regression analysis. A *P-*value less than 0.05 was considered statistically significant.

## Results

### Glucose Metabolism Disorders in Patients With Severe Hepatitis Flare of Chronic HBV Infection

Among 118 patients with severe hepatitis flare of chronic HBV infection, 70 patients had liver cirrhosis and 48 patients did not have liver cirrhosis. As shown in **Table 1**, in patients with liver cirrhosis, 33 (47.1%) and 25 (35.7%) patients had diabetes and IGT, respectively. In 48 patients without liver cirrhosis, 8 (16.8%) patients had diabetes and 23 (47.8%) patients had IGT. The prevalence of diabetes in patients with liver cirrhosis was higher than that in patients without liver cirrhosis (*P <*0.01), whereas prevalence of IGT in patients with liver cirrhosis was lower than that in patients without liver cirrhosis (*P <*0.01).

**Table 1:** Clinical characteristics and glucose metabolism disorders in patients with and without liver cirrhosis during severe hepatitis flare.

Variables	Total (n = 118)	Without LC (n = 48)	With LC (n = 70)	*P* value
Man (n%)	94 (79.7)	37 (77.1)	57 (81.4)	0.5651
Age (years)	43.4 ± 12.3	38.0 ± 12.1	47.2 ± 11.0	0.0002
BMI (kg/m^2^)	22.9 ± 2.6	23.2 ± 2.8	22.7 ± 2.4	0.3032
IGT (n%)	48 (39.0)	23 (43.8)	25 (35.7)	0.0071
Diabetes (n%)	41 (36.4)	8 (20.8)	33 (47.1)	0.0061
HOMA-IR	1.9 (1.2, 3.1)	1.8 (1.0, 3.0)	2.0 (1.4, 3.1)	0.1263
HOMA-IS	0.5(0.3, 0.8)	0.5 (0.3, 1.0)	0.5 (0.3, 1.0)	0.1263
HOMA-β	165.0 (110.4, 238.3)	190.7 (113.4, 264.0)	152.4 (110.0, 211.1)	0.3863
FPG (mmol/L)	5.0 ± 1.5	4.7 ± 0.9	5.2 ± 1.8	0.0822
2h-PG (mmol/L)	10.0 ± 3.4	8.9 ± 3.2	10.8 ± 3.3	0.0022
FINS (ulU/ml)	10.5 ± 5.7	10.1 ± 6.2	10.8 ± 5.3	0.5132
FCP (pmol/L)	841.7 ± 329.4	848.0 ± 373.3	837.3 ± 298.3	0.8632
INR	1.08 ± 0.22	0.96 ± 0.18	1.18 ± 0.20	0.0002
PTA (%)	91.5 ± 31.3	111.5 ± 28.7	77.7 ± 25.1	0.0002
ALT (U/L)	471.0 (265.0, 806.8)	712.0 (418.8, 888.5)	360.5 (78.3, 715.5)	0.0003
AST (U/L)	253.0 (136.3, 496.3)	366.0 (202.3, 546.3)	193.0 (93.0, 406.5)	0.0023
TBil (umol/L)	44.9 (25.2, 138.4)	40.9 (21.6, 169.3)	46.2 (27.3, 120.6)	0.7823
ALB (g/L)	35.6 ± 5.8	37.7 ± 4.6	34.2 ± 6.1	0.0002
Na^+^ (mmol/L)	138.2 ± 3.0	138.5 ± 2.2	138.0 ± 3.5	0.3922
Cr (umol/l)	74.9 ± 25.7	77.9 ± 35.8	72.8 ± 15.5	0.2942
UA (umol/l)	284.7 ± 85.5	300.3 ± 90.2	274.0 ± 81.1	0.1012
TG (mmol/L)	1.50 ± 0.80	1.72 ± 0.89	1.36 ± 0.70	0.0162
TC (mmol/L)	3.28 ± 0.90	3.39 ± 0.97	3.21 ± 0.85	0.3042
HDL (mmol/L)	0.81 ± 0.35	0.81 ± 0.38	0.81 ± 0.34	0.9652
LDL (mmol/L)	2.12 ± 0.64	2.27 ± 0.60	2.02 ± 0.64	0.0342
WBC (10^9^/L)	4.8 ± 1.7	5.5 ± 1.6	4.3 ± 1.5	0.0002
PLT (10^9/^L)	139.8 ± 64.0	186.0 ± 51.1	108.1 ± 51.5	0.0002
HBVDNA (lg copies/ml)	5.7 ± 2.0	5.7 ± 2.0	5.8 ± 2.0	0.9612
AFP (ng/ml)	17.1 (6.0, 84.8)	12.0 (5.5, 27.4)	26.6 (6.6, 130.4)	0.0093
MELD score	8.9 (6.0, 14.2)	7.2 (3.5, 13.9)	10.0 (7.1, 14.4)	0.0193
FIB-4	4.5 (2.3, 7.5)	2.6 (1.9, 4.8)	6.3 (3.7, 10.9)	0.0003
APRI	5.1 (2.6, 10.1)	4.9 (2.7, 9.1)	5.2 (2.5, 11.1)	0.6873

P < 0.05 was statistically significant; Data are presented as mean ± SD, n (%), or median (interquartile range). 2h-PG, 2-h post oral glucose tolerance test plasma glucose; AFP, alpha fetoprotein; ALB, albumin; ALT, alanine aminotransferase; APRI, aminotransferase-to-platelet ratio index; AST, aspartate aminotransferase; BMI, body mass index; Cr, creatinine; FCP, fasting C-peptide; FIB-4, fibrosis-4 score; FINS, fasting insulin; FPG, fasting plasma glucose; HBVDNA, hepatitis B virus DNA; HDL, high-density lipoprotein; HOMA-IR, homeostasis model assessment-insulin resistance index; HOMA-IS, homeostasis model assessment-insulin secretion index; HOMA-β, homeostasis model Assessment-islet beta cell function; IGT, impaired glucose tolerance; INR, international normalized ratio; LC, liver cirrhosis; LDL, low-density lipoprotein; MELD, model for end-stage liver disease; Na^+^, sodium; PLT, platelet; PTA, prothrombin activity; TBiL, total bilirubin; TC, total cholesterol; TG, triglyceride; UA, uric acid; WBC, white blood cell.

Additionally, patients with liver cirrhosis had an older age; higher levels of 2h-PG, INR, alpha fetoprotein (AFP), MELD score, and FIB-4 scores; and lower levels of triglyceride (TG), low-density lipoprotein (LDL), PTA, ALT, PLT, and white blood cells (WBC) than those in patients without liver cirrhosis.

### The Dynamic Changes in Glucose Metabolism Disorders in Patients Without Liver Cirrhosis

**Figure 1:**
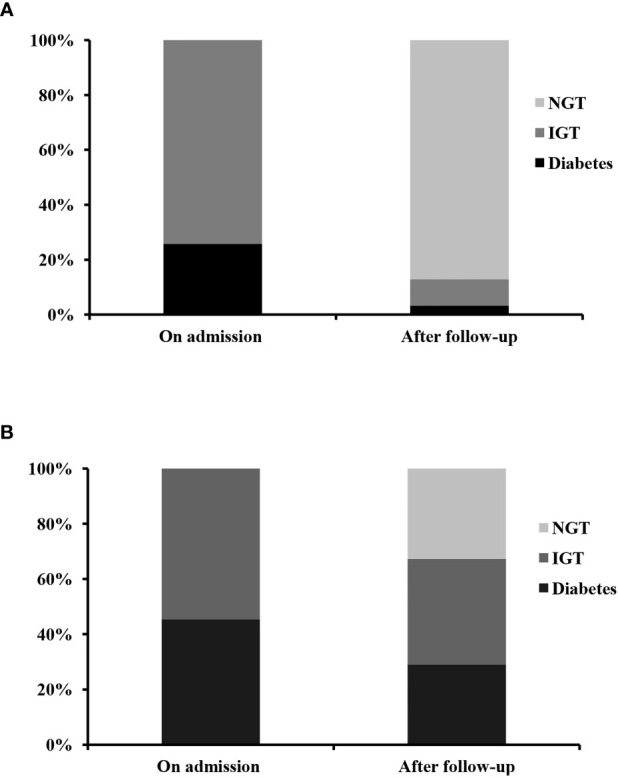
The dynamic change of glucose metabolism disorders in patients without **(A)** and with **(B)** liver cirrhosis during severe hepatitis flare of chronic hepatitis B virus infection. NGT, normal glucose tolerance; IGT, impaired glucose tolerance.

In 48 patients without liver cirrhosis, 31 patients had glucose metabolism disorders (23 patients with IGT and 8 patients with diabetes). All of them did not receive anti-hyperglycemic treatment and were followed up for 20.0 ± 18.7 days. As shown in **Table 2** and **Figure 1A**, at the end of follow-up, most patients had their liver function significantly improved. In 28 (90.3%) patients, glucose metabolism disorders regressed. In all, 27 (87.1%) patients experienced a regression of glucose metabolism disorders to NGT, including 6 patients with diabetes and 21 patients with IGT. One patient with diabetes regressed to IGT, and 2 patients with IGT remained unchanged. A significant decrease in 2-h post OGTT insulin (2h-Ins), 2-h post OGTT C-peptide (2h-PC), and 2h-PG was recorded by the end of follow-up, as compared with those on admission. However, by the end of follow-up, HOMA-β and HOMA-IR did not significantly differ from those on admission.

**Table 2:** The dynamic changes of glucose metabolism disorders and laboratory parameters in patients without liver cirrhosis.

Variables	On admission (n = 31)	After follow-up (n = 31)	*P* value
ALT (U/L)	766.0 (601.0, 951.0)	65.0 (36.0, 88.0)	0.000
AST (U/L)	425.0 (228.0, 719.0)	37.0 (29.0, 56.0)	0.000
TBil (umol/L)	56.0 (22.3, 171.1)	22.1 (14.5, 31.5)	0.000
ALB (g/L)	37.88 ± 4.49	38.67 ± 4.91	0.272
WBC (10^9^/L)	5.07 (4.32, 5.98)	5.30 (4.60, 6.06)	0.342
PLT (10^9^/L)	185.03 ± 46.29	209.58 ± 56.80	0.001
INR	0.89 (0.85, 1.04)	0.85 (0.80, 0.91)	0.015
PTA (%)	122.9 (98.0, 132.3)	133.0 (119.7, 149.5)	0.009
FPG (mmol/L)	4.77 (4.31, 5.11)	4.64 (4.31, 5.30)	0.906
FINS (ulU/ml)	7.70 (5.10, 14.30)	10.30 (6.50, 15.20)	0.112
FCP (pmol/L)	769.5 (534.9, 1101.0)	876.5 (700.7, 1141.0)	0.142
2h-PG (mmol/L)	9.14 (8.55, 11.13)	6.21 (5.25, 7.47)	0.000
2h-Ins (ulU/ml)	132.20 (46.7, 172.8)	62.10 (28.30, 104.90)	0.004
2h-CP (pmol/L)	4628.0 (2844.0, 5686.0)	3167.0 (2199.0, 4698.0)	0.002
HOMA-IR	1.75 (0.91, 3.24)	1.97 (1.19, 3.12)	0.210
HOMA-β	167.4 (84.8, 210.3)	197.9 (120.9, 250.7)	0.112
HBVDNA (lg copies/ml)	5.55 ± 1.85	2.81 ± 1.64	0.000
NGT (n%)	0	27 (87.1%)	0.000
IGT (n%)	23 (74.2%)	3 (9.7%)	0.000
Diabetes (n%)	8 (25.8%)	1 (3.2%)	0.000

P <0.05 was statistically significant; Data are presented as mean ± SD, n (%), or median (interquartile range); 2h-CP, 2-h post oral glucose tolerance test C-peptide; 2h-Ins, 2-h post oral glucose tolerance test insulin; 2h-PG, 2-h post oral glucose tolerance test plasma glucose; ALB, albumin; ALT, alanine aminotransferase; AST, aspartate aminotransferase; FCP, fasting C-peptide; FINS, fasting insulin; FPG, fasting plasma glucose; HBVDNA, hepatitis B virus DNA; HOMA-IR, homeostasis model assessment-insulin resistance; HOMA-β, homeostasis model assessment-β-cell function; IGT, impaired glucose tolerance; INR, international normalized ratio; NGT, normal glucose tolerance; PLT, platelet; PTA, prothrombin activity; TBiL, total bilirubin; WBC, white blood cell.

### The Dynamic Changes in Glucose Metabolism Disorders in Patients With Liver Cirrhosis

In patients with liver cirrhosis, 58 patients had glucose metabolism disorders. Fifty-five patients (25 patients with IGT and 30 patients with diabetes) were followed up for 42.0 ± 36.2 days. The other three patients were excluded, because they had received anti-hyperglycemic treatment during follow-up. As shown in **Table 3** and **Figure 1B**, after a follow-up of 42.0 ± 36.2 days, ALT, AST, glutamine transpeptidase (GGT), TBil, AFP, HBVDNA, 2h-PG, 2h-PC, FIB-4, and APRI levels had significantly decreased. Whereas albumin (ALB) and prealbumin (PA) levels had significantly increased, as compared with those on admission. Thirty (54.5%) patients experienced a regression of glucose metabolism disorders, including five patients with diabetes and 13 patients with IGT who regressed to NGT. Twelve patients with diabetes regressed to IGT. However, only 18 (32.7%) patients, including 5 patients with diabetes and 13 patients with IGT, experienced a regression of glucose metabolism disorders to NGT. By the end of follow-up, HOMA-β and HOMA-IR levels were not significantly different from those of patients on admission.

**Table 3:** The dynamic changes of glucose metabolism disorders and laboratory parameters in patients with liver cirrhosis.

Variables	On admission (n = 55)	After follow-up (n = 55)	*P* value
ALT (U/L)	235.0 (36.0, 795.0)	37.0 (28.0, 59.0)	0.000
AST (U/L)	173.0 (57.0, 408.0)	46.0 (35.0, 60.0)	0.000
GGT (U/L)	94.0 (47.0, 168.0)	55.0 (35.0, 96.0)	0.000
TBiL (U/L)	80.9 (38.6, 180.3)	35.0 (19.4, 58.1)	0.000
ALB (g/L)	30.90 ± 5.29	33.31 ± 4.63	0.019
PA (mg/L)	58.0 (36.0, 82.0)	112.0 (65.0, 154.0)	0.000
HBVDNA (lg copies/ml)	5.32 ± 2.37	2.27 ± 1.00	0.009
AFP (ng/mL)	33.59 (8.20, 136.03)	7.79 (5.30, 26.05)	0.006
PLT (10^9^/L)	97.0 (52.0, 149.0)	87.0 (56.0, 142.5)	0.881
FPG (mmol/L)	4.90 (4.21, 5.62)	4.93 (4.51, 5.86)	0.678
FINS (uIU/mL)	11.60 (7.10, 17.40)	11.50 (9.00, 18.00)	0.395
FCP (pmol/L)	961.5 (691.1, 1255.0)	900.9 (718.1,1093.0)	0.532
2h-PG (mmol/L)	11.24 (9.72, 13.20)	8.64 (7.33,11.56)	0.000
2h-Ins (uIU/mL)	118.0 (74.4, 216.3)	107.1 (87.9, 159.9)	0.140
2h-PC (pmol/L)	4269.4 ± 2015.1	3834.8 ± 1365.5	0.003
FIB-4	7.50 (3.59, 10.91)	4.05 (2.17, 8.07)	0.000
APRI	4.66 (2.34, 10.29)	1.40 (0.78, 2.16)	0.000
HOMA-IR	2.42 (1.57, 4.67)	2.85 (1.93, 4.67)	0.307
HOMA-β	172.8 (116.2, 294.3)	156.3 (118.2, 251.5)	0.542
NGT (n, %)	0	18 (32.7%)	0.000
IGT (n, %)	25 (45.5%)	21 (38.2%)	0.000
Diabetes (n, %)	30 (54.5%)	16 (29.1%)	0.000

P < 0.05 was statistically significant. Data are presented as mean ± SD, n (%), or median (interquartile range). 2h-CP, 2-h post oral glucose tolerance test C-peptide; 2h-Ins, 2-h post oral glucose tolerance test insulin; 2h-PG, 2-h post oral glucose tolerance test plasma glucose; AFP, alpha fetoprotein; ALB, albumin; ALT, alanine aminotransferase; APRI, aminotransferase to platelet ratio index; AST, aspartate aminotransferase; FCP, fasting C-peptide; FIB-4, fibrosis-4 score; FPG, fasting plasma glucose; FINS, fasting insulin; GGT, glutamine transpeptidase; HBV-DNA, hepatitis B virus DNA; HOMA-IR, homeostasis model assessment-insulin resistance; HOMA-β, homeostasis model assessment-β-cell function; IGT, impaired glucose tolerance; INR, international normalized ratio; NGT, normal glucose tolerance; PA, prealbumin; PLT, platelet; PTA, prothrombin activity; TBiL, total bilirubin.

### The Factors Associated With the Regression of Glucose Metabolism Disorders in Patients With Liver Cirrhosis

As shown in **Table 4**, patients with liver cirrhosis who had regression of glucose metabolism disorders had higher levels of HOMA-β and ALB and a lower level of FIB-4 than those without regression. ALB (OR=1.238, 95% CI: 1.046~1.466) was identified as an independent factor associated with the regression of glucose metabolism disorders in patients with severe hepatitis flare of liver cirrhosis (**Table 5**).

**Table 4:** Glucose metabolism and laboratory parameters in patients with and without regression of glucose metabolism disorders by the end of follow-up.

Variables	Regression (n = 30)	No-regression (n = 25)	*P* value
ALT (U/L)	43.0 (30.8, 60.0)	35.0 (21.0, 56.0)	0.250
AST (U/L)	49.7 ± 16.3	46.0 (33.5, 60.0)	0.593
TBil (umol/L)	24.2 (17.9, 42.8)	44.7 (20.5, 60.9)	0.055
ALB (g/L)	34.0 (31.9, 38.4)	30.2 (28.5, 34.0)	0.007
PA (mg/L)	127.4 ± 59.5	102.2 ± 65.5	0.612
AFP (ng/mL)	7.04 (5.14, 11.13)	12.71 (5.47, 49.44)	0.235
FPG (mmol/L)	4.93 (4.50, 5.62)	5.06 (4.79, 6.09)	0.059
FINS (uIU/mL)	11.45 (8.85, 19.53)	11.50 (9.00,16.85)	0.710
FCP (pmol/L)	896.6 (718.8, 1133.3)	900.9 (715.6, 1121.0)	0.852
2h-Ins (uIU/mL)	107.30 (95.70, 154.60)	105.00 (62.65, 178.70)	0.694
2h-CP (pmol/L)	3864.56 ± 1236.91	3782.36 ± 1537.43	0.203
HOMA-IR	2.41 (1.76, 4.51)	2.95 (2.07, 4.76)	0.510
HOMA-β	197.70 (136.01, 315.71)	132.15 (64.31, 171.78)	0.004
FIB-4	2.78 (1.81, 6.79)	6.26 (2.50, 10.07)	0.050
APRI	1.18 (0.73, 1.89)	1.49 (0.80, 2.61)	0.236

P < 0.05 was statistically significant. Data are presented as mean ± SD, n (%), or median (interquartile range). 2h-CP, 2-h post oral glucose tolerance test C-peptide; 2h-Ins, 2-h post oral glucose tolerance test insulin; AFP, alpha fetoprotein; ALB, albumin; ALT, alanine aminotransferase; APRI, aminotransferase to platelet ratio index; AST, aspartate aminotransferase; FCP, fasting C-peptide; FIB-4, fibrosis-4 score; FINS, fasting insulin; FPG, fasting plasma glucose; HOMA-IR, homeostasis model assessment-insulin resistance; HOMA-β, homeostasis model assessment-β-cell function; PA, prealbumin; TBiL, total bilirubin.

**Table 5:** Univariate and multivariate analyses of factors associated with regression of glucose metabolism disorders in patients with liver cirrhosis.

Variables	Univariate analysis				Multivariate analysis			
	B	OR	95%CI	*P*	B	OR	95%CI	*P*
ALB	0.214	1.238	1.046~1.466	0.013	0.214	1.238	1.046~1.466	0.013
HOMA-β	0.008	1.008	1.002~1.041	0.014				
FIB-4	0.116	1.123	0.976~1.292	0.105				

ALB, albumin; CI, confidence interval; FIB-4, fibrosis-4 score; HOMA-β, homeostasis model assessment-β-cell function; OR, odds ratio.

## Discussion

Patients with hepatitis flare of chronic HBV infection have different degrees of acute and chronic liver injury and inflammation, which may trigger glucose metabolism disorders. As a result, the clinical manifestations and pathogenesis of glucose metabolism disorders are more complex than those of liver cirrhosis or acute hepatitis. The dynamic changes and the factors associated with regression of glucose metabolism disorders in patients with hepatitis flare of chronic HBV infection have yet to be studied. In this study, we excluded patients with ACLF because patients with ACLF had distinct pathogenesis with potential effect on glucose metabolism (Kumar et al., 2020). The major findings of this study are that the severe hepatitis flare mostly resulted in IGT in patients without liver cirrhosis, whereas it mostly resulted in diabetes in patients with liver cirrhosis. After acute liver inflammation resolved, most patients without liver cirrhosis experienced a regression of the glucose metabolism disorders. However, only about 50% of patients with liver cirrhosis experienced a regression of glucose metabolism disorders. High ALB level was associated with the regression of glucose metabolism disorders in patients with liver cirrhosis and severe hepatitis flare.

Glucose metabolism disorders in patients with acute liver inflammation have been evaluated in several previous studies (Del Vecchio Blanco et al., 1990; Buzzelli et al., 1998). However, due to the diversity in etiology, diagnostic methods of glucose metabolism disorders, and severity of liver inflammation in study participants, different prevalence rates of glucose metabolism disorders have been reported. In this study, we found that in patients without liver cirrhosis, 16.8% and 47.8% of patients had diabetes and IGT, respectively, which are significantly higher than those in the general population in China (Ma, 2018). We also found that 28 of 31 (90.3%) patients without liver cirrhosis experienced regression of glucose metabolism disorders after 20.0 ± 18.7 days of follow-up. These results demonstrated that severe acute liver inflammation and acute liver injury only triggered temporary glucose metabolism disorders, mostly resulting in IGT.

Several studies have investigated the prevalence and clinical significance of diabetes in patients with liver cirrhosis. The etiology and severity of liver cirrhosis have been reported to be related to the occurrence of HD (Nishida, 2017; Lee W. G., et al., 2019). However, most previous studies did not study the effects of acute liver inflammation on the prevalence and severity of glucose metabolism disorders in patients with liver cirrhosis. In this study, we found that glucose metabolism disorders regressed after acute liver inflammation resolution in 30 of 55 patients with liver cirrhosis, demonstrating that severe acute liver inflammation aggravated glucose metabolism disorders in patients with liver cirrhosis. These results suggest that in evaluating glucose metabolism disorders in patients with liver cirrhosis, the effect of acute liver inflammation must be considered.

The pathogenesis of HD and stress-induced hyperglycemia remains to be elucidated. Previous studies have found that both pancreatic β cell dysfunction and increased insulin resistance play a central role in the development of HD in patients with liver cirrhosis (Grancini et al., 2015). Additionally, stress-induced hyperglycemia is considered to be due to pancreatic failure to compensate for increasing insulin production and release (Mifsud et al., 2018). The factors associated with the regression of glucose metabolism disorders in patients with acute and chronic liver diseases remain unclear. Grancini et al. (2019) found that, after liver transplantation, the pancreatic β cell function determined the regression of glucose metabolism disorders. Although in this study we found that patients without regression of glucose metabolism disorders had a significantly lower HOMA-β level than patients with regression of glucose metabolism disorders, only ALB was the independent factor associated with the regression of glucose metabolism disorders. This discrepancy is, however, difficult to explain; the difference in study participants may be a possible explanation. In this study, only patients with liver cirrhosis and severe acute liver inflammation were included; patients with ACLF were excluded. Furthermore, we only observed the short-term regression of glucose metabolism disorders. However, according to the study by Grancini et al. (2019), all patients had more advanced liver cirrhosis and most patients had ACLF. Moreover, their results were obtained after 24 months of follow-up. Since a higher ALB level might reflect less degrees of liver fibrosis or cirrhosis (Tsochatzis et al., 2014), our results suggest that less degrees of liver fibrosis or cirrhosis causes short-term regression of glucose metabolism disorders in patients with severe hepatitis flare and liver cirrhosis after acute liver inflammation resolved.

Although several studies have reported that hyperglycemia is associated with high mortality in patients with liver cirrhosis and critically ill patients, the management of hyperglycemia in these patients remains a clinical challenge. Hyperglycemia increases mortality in patients with liver cirrhosis and critically ill patients. However, strict control of hyperglycemia might increase the prevalence of hypoglycemia, which would be associated with high mortality (Ruan et al., 2019; Kushimoto et al., 2020). Therefore, understanding the dynamic change in glucose metabolism disorders in patients with hepatitis flare of chronic HBV infection is clinically significant. In this study, we found that most patients without liver cirrhosis experienced a regression of glucose metabolism disorders as liver inflammation resolved. However, in patients with liver cirrhosis, only about one-third of patients experienced a regression of glucose metabolism disorders. This suggests that glucose metabolism should be evaluated in patients with liver cirrhosis after the resolution of acute liver inflammation, especially in patients with sustained low levels of ALB.

Our study had several limitations. First, although this study used the largest cohort to study the dynamic change of glucose metabolism disorders in patients with severe hepatitis flare of chronic HBV infection by performing OGTT, it was a single center-based study. The number of patients included in this study was relatively small. Therefore, the study findings may still need to be verified in multi-center studies with larger sample sizes. Second, only short-term dynamic changes in glucose metabolism disorders were evaluated. Long-term changes in glucose metabolism disorders remain to be clarified in future studies. Third, only changes in glucose metabolism disorders in patients with resolution of liver inflammation were evaluated, the changes in glucose metabolism disorders in patients with aggravation of liver inflammation and their associations with disease progression remain to be determined.

In conclusion, this study is the first to evaluate short-term dynamic changes of glucose metabolism disorders in patients with hepatitis flare of chronic HBV infection. Resolution of acute liver inflammation resulted in a regression of glucose metabolism disorders in most patients without liver cirrhosis. Whereas few patients with liver cirrhosis experienced a regression of glucose metabolism disorders. High ALB level was associated with regression of glucose metabolism disorders in patients with liver cirrhosis.

## Data Availability Statement

The original contributions presented in the study are included in the article/supplementary material. Further inquiries can be directed to the corresponding author.

## Ethics Statement

The studies involving human participants were reviewed and approved by Human Ethical Committee of the Affiliated Hospital of Zunyi Medical University. The patients/participants provided their written informed consent to participate in this study.

## Author Contributions

SL designed the experiments and drafted the manuscript. CT, YPZ, YuL, HH, QC, YHZ, and LP performed the experiments and collected the data. YiL and FY revised the manuscript and analyzed the data. All authors read and approved the final manuscript.

## Funding

This work was supported by funding from the Chinese National Natural Science Foundation Project (81860114). The funders had no role in the study design and analysis, decision to publish, or preparation of the manuscript. No additional external funding was received for this study.

## Conflict of Interest

The authors declare that the research was conducted in the absence of any commercial or financial relationships that could be construed as a potential conflict of interest.

## Publisher’s Note

All claims expressed in this article are solely those of the authors and do not necessarily represent those of their affiliated organizations, or those of the publisher, the editors and the reviewers. Any product that may be evaluated in this article, or claim that may be made by its manufacturer, is not guaranteed or endorsed by the publisher.
